# Hybrid Materials Based on Carbon Nanotubes and Nanofibers for Environmental Applications

**DOI:** 10.3389/fchem.2020.00546

**Published:** 2020-06-30

**Authors:** Anastasiya G. Navrotskaya, Darya D. Aleksandrova, Elena F. Krivoshapkina, Mika Sillanpää, Pavel V. Krivoshapkin

**Affiliations:** ^1^ChemBio Cluster, ITMO University, Saint Petersburg, Russia; ^2^Institute of Research and Development, Duy Tan University, Da Nang, Vietnam; ^3^Faculty of Environment and Chemical Engineering, Duy Tan University, Da Nang, Vietnam; ^4^Faculty of Health, Engineering and Sciences, School of Civil Engineering and Surveying, University of Southern Queensland, Toowoomba, QLD, Australia

**Keywords:** carbon nanotubes, carbon nanofibers, hybrid nanomaterials, inorganic nanoparticles, environmental application

## Abstract

With the advances in material science, hybrid nanomaterials with unique mechanical, electrical, thermal and optical characteristics have been developed. Among them, hybrids based on filamentous forms of carbon, such as carbon nanotubes and carbon nanofibers, in combination with inorganic nanoparticles attract particular attention. Due to the structure and morphology, charge and energy transfer processes lead to synergistic effects that allow the use of less material with higher productivity. To clarify these issues, this review will summarize and discuss the relevant studies of the use of inorganic compounds of various chemical groups in modifying carbon nanomaterials for ecological applications.

## Introduction

Carbon based materials have a number of different properties, and today, are used in all areas of life, including industry, metallurgy, medicine, optics, and environmental protection. However, the rapid development of industries demands more advanced materials with new characteristics created for future uses. The solution was found in the creation of hybrid materials that not only combine the properties of individual components, but also lead to synergistic effects.

Briefly, hybrid materials (HMs) are a result of mixing chemically different components with the formation of interactions, such as Van der Waals, hydrogen bonding, weak electrostatic interactions or covalent bonds. When formed, HMs have a structure different from that of their component materials, but inherit some of their properties and functions. The important factor is the inner structure of the hybrid. By manipulating this aspect, we can control the physicochemical properties of the hybrid material. Combination of carbon nanomaterials (CNMs) with polymers and inorganic nanoparticles improves mechanical (Gomathi et al., 2005; Zhao et al., 2011; Dillon et al., 2015; Wu et al., 2017), electrical (Whitsitt and Barron, 2003; Hang et al., 2005; Ivnitski et al., 2008; Liang et al., 2012), thermal (Cui et al., 2011; Chen L. et al., 2014; Aghabozorg et al., 2016; Hameed et al., 2019), sorptive (Deng et al., 2005; Choi et al., 2010; Czech et al., 2015; Saud et al., 2015; Navrotskaya et al., 2019) and catalytic (Wu et al., 2009; Paula et al., 2011; Aazam, 2014; Kim et al., 2014) properties(Kumar et al., 2008; Wu et al., 2009; Cui et al., 2011; Dillon et al., 2015).

**Figure 1:**
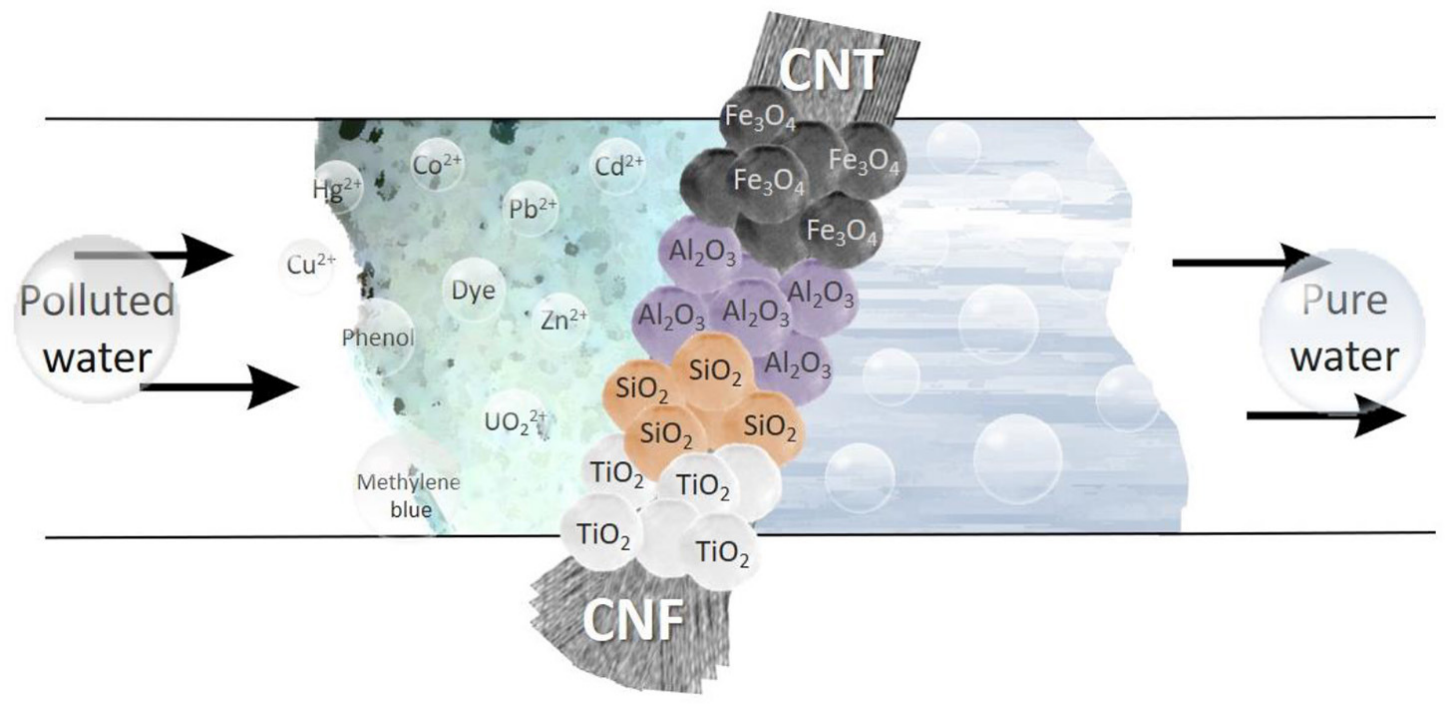
Creation and application of hybrid materials.

Thus, currently there emerges an opportunity to modify CNMs with various nanomaterials using elements of the periodic table, namely metal and metal oxide nanoparticles and inorganic salts. In this context, this review summarizes recent progress in the fabrication and utilization of hybrid materials based on carbon nanomaterials and inorganic nanoparticles. It is especially worth noting that carbon structures, such as graphite, diamond, glassy carbon, graphene, amorphous powders, carbon fibrous materials, carbon nanofibers (CNFs), and carbon nanotubes (CNTs), are very interesting materials for research, development and large-scale production. One of the many advantages of CNTs and CNFs is their length to width ratio (>1,000), which results in a filamentous structure which translates to a high specific surface area (Wu et al., 2009; Paula et al., 2011; Aazam, 2014; Kim et al., 2014). In this regard, this review focuses solely on the advances of hybrid materials based on CNFs and CNTs for environmental applications, which distinguishes it from a number of works dedicated to carbon nanomaterials (Figure 1).

## Carbon Nanomaterials

### Carbon Nanotubes

Carbon nanotubes were first reported by Radushkevich and Lukyanovich in 1952 (Thakur and Thakur, 2016) and scientifically reported by Iijima in 1991 (Iijima, 1991). Carbon nanotubes are a seamless cylindrical graphene layer with half of a fullerene molecule at each end (Sarkar et al., 2018; Vashist et al., 2018a,b). CNTs are several nanometers in diameter, but several millimeters in length (Thakur and Thakur, 2016). Depending on the number of layers, CNTs can be single-walled (single-layer) (SWCNTs) or multi-walled (multi-layer) (MWCNTs) (Dai, 2002; Aqel et al., 2012; Das et al., 2014; Postnov et al., 2016). MWNTs are more attractive for widespread use as they are cheaper than SWCNTs (Aqel et al., 2012; Liu et al., 2013; Postnov et al., 2016; Thakur and Thakur, 2016). MWCNTs are made up of several concentric graphene pipes. Individual layers may be described as SWCNTs, which can be a semiconductor or metal. CNTs have a porous structure (Zeng et al., 2014; Chen et al., 2015; Zaytseva and Neumann, 2016).

Carbon nanofibers (CNFs) are filamentous nanomaterials that have mechanical and electrical properties similar to CNTs (Bergmann and Machado, 2015). There are, however, some key differences. Firstly, CNFs are not hollow. Also, the structure of CNFs can be described as graphene layers arranged perpendicularly or at an angle to the fiber axis (Klein et al., 2008; Mishakov et al., 2008; Feng et al., 2014; Yan et al., 2015). The most common CNF structures are “stack of coins” (or plane-parallel, “stacked”), “Christmas-tree structure” (or stack of cones, “fishbone,” coaxial-conical), and “stack of cups” (or “lampshades,” “bamboo”) (Klein et al., 2008).

The similar structure of CNFs is due to their growing mechanism, which depends on the geometric characteristics of metal catalyst particles and the carbon source gas (Poveda and Gupta, 2016). CNFs are about several micrometers in length and have diameters ranging from 5 to 200 nm (Huang et al., 2010; Feng et al., 2014). Ballistic electron transport and tensile strength along the axis, as in diamond, are inherent characteristics of CNTs. CNFs, on the other hand, have higher reactivity and electron transfer through the sidewalls, which is important for functionalization and electrochemical application, respectively (Klein et al., 2008).

### Purification and Functionalization of CNTs and CNFs

One of the stages of hybrid materials preparation is removing amorphous carbon, fullerenes, and metal catalyst particles from the CNFs and CNTs surface (Eder, 2010). There are several purification methods, each with its own advantages and disadvantages. Process efficiency should be the main criterion when choosing a purification method. It depends on the purity of the starting material, time and temperature of oxidation, pH and oxidizing agent. For example, carbon impurities can be removed via oxygen treatment, which is simply passing an H_2_S and O_2_ air mixture over the CNFs and CNTs. However, oxidation often results in broken surface tubes or fibers, especially when combined with ultrasonic and high-temperature processing. Oxidation via strong acids, such as HNO_3_, H_2_SO_4_, another purification method, leads to broken surface tubes or fibers, as well as the formation of various functional groups. For the removal of metal catalyst particles without interfering with the carbon nanostructure, non-oxidizing acid treatment (for example HCl) is usually used. This leads to the fact that the metal nanoparticles move into the solution and leave the nanosystem. As an alternative to the above methods, high-temperature annealing in vacuum or inert gas can be performed. The processing temperature depends on the purpose and ranges from 600 to 2,000°C.

Much research has been dedicated to the surface functionalization of nanotubes for the creation of new materials with unique properties. This implies that CNTs are treated with different substances to form different functional groups on the surface (Thakur and Thakur, 2016). Covalent functionalization occurs when a covalent bond is formed between the carbon surface and the modifying agent. Functional groups can form at the end or on a sidewall of the nanotubes and nanofibers. For a single-walled carbon nanotube, this type of functionalization can lead to a shift in the electronic structure and thereby affect the conductivity. In the case of multi-walled carbon nanotubes, the internal electronic structure is preserved and new surface characteristics appear, which expands the possibilities of their application (Thakur and Thakur, 2016). In fact, covalent functionalization is carried out by organic molecules that interact with carboxyl groups after surface oxidation (Bright, 2000; Sahoo et al., 2010; Gao et al., 2012; Rabti et al., 2016).

Another type of functionalization—namely non-covalent functionalization—arises through Van der Waals forces and hydrogen bonding (Eder, 2010). Unlike covalent functionalization, it one does not lead to numerous surface defects or to any changes in the mechanical and conductive properties. In this case, modifying agents are various active substances and polymers that increase the solubility of CNTs in hydrophilic solvents and their dispersion in a polymer or ceramic matrix. Aromatic compounds (porphyrins, pyrenes) can also be included here due to π-π–interaction with the delocalized electron cloud of CNTs. The high curvature of CNTs determines reactivity connecting with π-orbital mismatch. The nanotube end, the fullerene hemispheres, are more reactive than the sidewalls. These properties can be used for the selective functionalization of CNTs.

Articles (Bright, 2000; Sahoo et al., 2010; Gao et al., 2012; Rabti et al., 2016) pay special attention to the positive influence of CNT surface modification. Through this process, the metal catalyst particles enter the solution in the form of salt and leave the nanosystem (Rao et al., 2007). In addition, the surface modification of carbon nanomaterials can lead to the formation of hydroxyl, carbonyl and carboxyl groups (Yang et al., 2009; Zawisza et al., 2012), and is most effectively achieved when the nanotubes (as sorbent) are oxidized using NaOCl, HNO_3_ and KMnO_4_ (Rao et al., 2007; Ihsanullah et al., 2016).

Carbon nanofibers can also be subjected to surface functionalization, but (unlike CNTs) their entire surface can be modified. CNFs activation by nitric acid or electrochemical oxidation can be used to form oxygen-containing groups without degradation of CNFs structure (Huang et al., 2010).

## Synthesis of Hybrid Materials

Inorganic hybrids based on CNTs and CNFs can be synthesized via *ex situ* and *in situ* methods. The first of these involves the separate preparation of the inorganic component in the desired size and morphology (usually spherical nanoparticles), then the attachment of this component to the carbon surface through covalent, non-covalent or electrostatic interactions. On the contrary, the *in situ* method involves the synthesis of an inorganic component in the presence of initial or functionalized CNTs and CNFs, on which the component grows in the form of particles, nanowires, or thin films (Eder, 2010). Filling the inside of a CNT with inorganic compounds from the gas or liquid phase is carried out by capillary forces based on condensation or wetting.

The valuable advantages of hybrid materials are the variety of synthesis routes and their relative simplicity. These materials can be obtained at low temperatures, through sol-gel and hydrothermal reactions, as well as in various morphologies, for example, in the form of three-dimensional structures, thin films or nanoparticles. The choice of methods for the synthesis of inorganic hybrids based on CNTs and CNFs and the degree of their synergistic effect depend on the type and purity of carbon materials, as well as their surface functionalization.

### Sol-Gel Method

Sol-gel method is nowadays a common practice and can be said to be a comparatively new type of synthesis. This synthesis method results in the uniformed distribution of inorganic particles on the surface of the carbon nanomaterials. This process is diffusion-controlled, and the changing pH causes precursors to polymerize and form the inorganic particles. Different types of inorganic coatings can be created depending on the precursors used. For example, the hydrolysis of titanium isopropoxide resulted in a titanium dioxide matrix (Kim et al., 2011; Li et al., 2011; Hamid et al., 2014; Ge et al., 2015); iron (III) nitrate–iron (III) oxide matrix (Sun et al., 2005, 2018; Hassan et al., 2013; Wan et al., 2015); and also probably the creation of ZrO_2_, HfO_2_, and Ta_2_O_5_ oxide gels (Miller and Ko, 1996; Benad et al., 2018; Kiselev et al., 2019). Fixing elemental oxide on CNTs or CNFs surfaces changes hybrid materials characteristics.

### Hydrothermal Treatment

Hydrothermal (and solvothermal) synthesis is conducted with a special piece of equipment called an autoclave under fixed pressure and temperature. Reagents are loaded into the autoclave then left in the oven for a period of time, allowing the reaction to take place without direct supervision (Byrappa and Adschiri, 2007; Yoshimura and Byrappa, 2008; Baruah and Dutta, 2009). During hydrothermal synthesis, aqueous solvents or mineralizers work under temperature and pressure to dissolve and recrystallize usual insoluble materials and decompose or recycle any waste material (Byrappa and Yoshimura, 2013). This process is carried out at high temperatures. As the precursors are the same as with the sol-gel synthesis method, hydrolysis is possible (Pirajno, 2009; Byrappa and Yoshimura, 2013). In this study, synthesized core-shell-structured carbon nanofiber (CNF)-titanate nanotubes (TiNT) by alkaline hydrothermal treatment. The CNF core could act as a support, and the TiO_2_-decorated TiNT shell could act as a photocatalyst. The surface area increase as a result of the alkaline hydrothermal treatment may be responsible for the efficient photocatalytic activity of CNF-TiNTs (Kim et al., 2014; Kong et al., 2014; Guo et al., 2019).

### Chemical Vapor Deposition (CVD) on Catalyst Nanoparticles

This method is often used in the semiconductor industry to obtain high clearing solid materials or thin films. Typically, during CVD, the substrate (catalyst) is placed in the precursor vapor and then the reaction produces the necessary substance. This process is used to obtain clean CNMs by making CNTs and CNFs then removing them from the nanoparticle-catalyst (substrate) surface (Bhat, 2006; Kumar and Ando, 2010; Prasek et al., 2011; Zhang et al., 2013; Bauman et al., 2017).

Nanomaterials can be used with the catalyst particles without separation. This resulting material is a hybrid. Here, catalyst particles act as both a substrate under the growing carbon nanomaterials, and as an arming dopant (Lee et al., 2002; Nessim, 2010). For use in ecology or the medical industry, catalysts must be non-toxic or must decrease the toxicity of carbon nanomaterials (Yu et al., 2011; Cendrowski et al., 2014; Chen J. et al., 2014).

In their publication, Cao et al. (2003) use this method to control the position and growth of CNTs (their length and direction) on the plane. Nanotube bridges connect samples of SiO_2_ and demonstrate good electrical properties. It is important to note that SiO_2_ neither coats CNTs nor decreases the conductivity. This method seems simple, inexpensive and controlled. Synthesized nanowires with dielectric shells present a new possibility for the effective and simple creation of high-pressure vertical broadband devices (Li et al., 2007).

Growing nanofibers on sphere nanoparticles leads to a significant increase in the fiber surface area. The reaction of growing CNTs decreases fiber strength, but the fiber module significantly increases, with compounds having grown CNTs fibers exhibiting significant improvement (up to 150%) in apparent shear strength in the transverse direction (Qian et al., 2010). The idea of hybridizing CNTs and Al_2_O_3_ is based on agglomeration prevention of CNTs due to Van der Waals interaction. Epoxide compounds with CNTs-Al_2_O_3_ demonstrate magnification >100% of compressive strength and Young's modulus (Zakaria et al., 2016). The introduction of nanocatalysts by this method is designed to improve the thermal properties of CNMs (Kumar et al., 2008; Ahmad et al., 2009, 2010).

### Electrostatic Self-Assembly

This method is realized due to the interaction of the charged of particles on surface charged substrate, resulting in strong bond formation and uniformed distribution (Fang and Böhringer, 2008; Liu Y. et al., 2009; Olmedo et al., 2011; Choi et al., 2014). One-dimensional nanocomposite colloids are prepared through electrostatic self-assembly of CdTe nanocrystals on both carbon nanotubes (CNTs) and silica coated CNTs. The dense coverage of these linear nanoparticle assemblies minimizes the spacing between the nanocrystals, thereby facilitating efficient electron and energy transfer along the nanotubes (Grzelczak et al., 2006; Bogani et al., 2009; Liu Y. et al., 2009; Downes et al., 2015).

## Hybrid Materials for Environmental Applications

Hybrid carbon nanomaterials are used in many areas of our life, such as medicine, material science, and environmental concerns. These are not the only areas, but, due to the main properties of CNMs, the nanosystems would be most effective in the aforementioned fields due to the synergetic effect (Table 1). In the current climatic conditions, the environmental situation is such that there is a rising demand to protect the environment from toxic substances. Pollution, the release of harmful substances into the environment, is one of the results of the human lifestyle. The huge release of copper, mercury and other trace elements has produced a list of complex environmental problems. These materials are likely toxic to all living organisms. Highly sensitive and selective results show that these substances have received considerable attention in the last few years (Ghiasvand et al., 2020). Removal of these compounds is a mandatory step in protecting the environment. This topic has interested many scientists from around the world (Song et al., 2010; Ashrafi et al., 2014; Sareen et al., 2014; Zare et al., 2015).

**Table 1:** Summary of the efficiency of various hybrid materials.

**Hybrid material**	**Toxic substances**	**Sorption capacity**	**References**
CNTs-Sb	Pb^2+^, Cd^2+^	37.50 ng/g, 0.34 μg/g	(Ashrafi et al., 2014)
CNTs-AgOH	${\text{UO}}_{2}^{2+}$	140 mg/g	(Zare et al., 2015)
CNTs sheets	Pb^2+^, Cd^2+^, Co^2+^, Zn^2+^, Cu^2+^	117.65, 92.59, 85.74, 74.63, 64.93 mg/g	(Tofighy and Mohammadi, 2011)
CNTs-Ni	Methylene blue	312 mg/g	(Jin et al., 2018)
CNTs-SiO_2_/Al_2_O_3_	NaCl	6.5 mg/g	(Santos et al., 2018)
CNFs-Fe_2_O_3_, CNTs-Fe_2_O_3_	Phenol	1.684, 2.778 mg/g	(Asmaly et al., 2015)
CNTs-Cu-BDC MOFs	Bisphenol A	164.1 mg/g	(Ahsan et al., 2019)
BN/rCNT	S	43 mg/g	(Xia et al., 2019)
CNF-GnP	Methylene blue, Congo red	1178.5. and 585.3 mg/g	(Yu et al., 2020)
PHO-CNF	U (VI)	1550.0 mg/g	(Lehtonen et al., 2020)
**Hybrid material**	**Dye**	**Photocatalytic activity**	**References**
CNTs-TiO_2_	Reactive Black 5	90%/15 min	(Hamid et al., 2014)
CNTs-TiO_2_-SiO_2_	Bisphenol A, carbamazepine	50%/30 min	(Czech and Buda, 2015)
CNFs-Fe_3_O_4_	Methylene blue, Rhodamine B (RhB)	95%/15 min	(Ren et al., 2012; Si et al., 2012)
CNFs-TiO_2_-ZnO	Methylene blue	40%/15 min	(Pant et al., 2013)
CNTs-PbBiO_2_Br	Ciprofloxacin	50%/30 min	(Wang B. et al., 2019)
CNTs-MoS_2_/SnS_2_	Cr (VI)	100%/90 min	(Dong et al., 2019)
CNTs-CoSnS	Rhodamine B	91.7%/80 min	(Jeyagopal et al., 2020)
CNFs-Cu	Chlortetracycline hydrochloride	68.2%/60 min	(Wang H. et al., 2019)

The important area is removing divalent heavy metal ions Cu^2+^, Zn^2+^, Pb^2+^, Cd^2+^, Co^2+^ from aqueous solutions. Pure CNTs (Tofighy and Mohammadi, 2011) and CNFs (Zheng et al., 2014) can be used as sorption agents. Preference of adsorption onto the oxidized CNT sheets can be ordered as Pb^2+^ > Cd^2+^ > Co^2+^ > Zn^2+^ > Cu^2+^ (Tofighy and Mohammadi, 2011). In the research of Asmaly et al. (2015), adsorption capacities increase in a row CNFs, untreated CNTs, CNFs-Fe_2_O_3_. The maximal sorption capacity has a material CNTs-Fe_2_O_3_. In their research Dr. Bagheri et al. propose a CNTs-magnetic SiO_2_ compound for finding Cu^2+^ and Hg^2+^, detectable even by human eyes (Li et al., 2007; Khani et al., 2010; Song et al., 2010; Bagheri et al., 2011; Ganjali et al., 2011). Because of its widespread use in modern society, copper poses serious environmental problems and is potentially toxic to all living organisms. Highly sensitive and selective detection of Cu^2+^ or Cu^+^ has received much attention in recent years.

The environmental impact of uranium and its associated health effects on humans has recently become a major concern—mainly due to the use of weakened uranium in armor-piercing bullets (Konstantinou et al., 2013). Radioactive uranium (VI) is weakened and loaded unto silver hydroxide nanoparticles—MWCNTs, which have been identified as an excellent adsorbent for the removal of ${\text{UO}}_{2}^{2+}$ ion from aqueous solutions (Zare et al., 2015). In this technique, the application of an ultrasonic wave during the synthesis of these nanomaterials led to properties, such as high surface area; enhanced removal percentage and high adsorption capacity; a high number of active centers; and a large number of vacant, available reactive surface sites in addition to metallic or semi-metallic behavior necessary for removal of various toxic materials (Fasfous and Dawoud, 2012; Sun et al., 2012; Chen et al., 2013; Tan et al., 2015).

Photocatalytic or adsorptive removal of organic pollutants has often been based on the example of phenol, that propagates to other toxic, organic, aromatic poisons (Ren et al., 2012; Si et al., 2012; Asmaly et al., 2015; Tho et al., 2018). Also, it can be methylene blue (Kim et al., 2011; Yu et al., 2011; Saud et al., 2015; Yu et al., 2015; Tho et al., 2018), 4-chlorophenol (Liu H. et al., 2009; Ihsanullah et al., 2015; Zouzelka et al., 2016), Remazol Black Brilliant (Shakouri et al., 2016), visible-light photocatalytic activity in the degradation of Rhodamine B (RhB) (Shang et al., 2013; Jiang et al., 2015), bisphenol A and carbamazepine (Czech and Buda, 2015), acetaminophen (Czech and Buda, 2015). In the submitted article (Ivnitski et al., 2008) nanocomposite CNT-TiO_2_/SiO_2_ was synthesized using the sol-gel method. Up to a 2.2 eV decrease in the bandgap was observed in the resulting material. Composites containing 8 mass % CNT exhibited maximum photoactivity. This article (Whitsitt and Barron, 2003) illustrates the decreased toxicity of this material. There is no limit to a number of components for a potential hybrid material. For example, TiO_2_/CCNFs (Graphene/carbon composite nanofibers) TiO_2_/ZnO/CNFs, CdS/TiO_2_/CNFs, Ag-AgI-TiO_2_/CNFs in articles (Kim et al., 2012; Pant et al., 2013, 2014; Yu et al., 2015), respectively show multicomponent hybrid materials. The composites showed high adsorption and photocatalytic activity under irradiation due to the synergetic effect between high adsorption ability, good conductivity of CNMs, and extraordinary plasmonic effect of nanoparticles.

## Future Directions and Concluding Remarks

Today, the scientific community has obtained promising results in the filamentous carbon based hybrid materials area. Hybrid materials are unique in that their properties are not the sum of the properties of the individual components, but their synergy. The hybrid structure provides an additional degree of freedom, which when developing new materials can lead to the emergence of new or improved properties (conductivity, sorption, catalytic, mechanical, optical, and magnetic properties). Currently, the problem of environmental protection remains one of the most urgent in the world. Hybrids based on carbon nanotubes and carbon nanofibers in combination with inorganic (metal oxide) nanoparticles can potentially solve the problems of water and air pollution, and recycling. With them being highly efficient sorbents and photocatalysts, higher productivity can be seen using less material. Therefore, this area of the research has high potential in the development of high-performance materials. Meanwhile, future work toward obtaining the compatibility between carbon nanomaterials and functional nanomaterials is essential to advance the use of these hybrids in electronic, magnetic and environmental applications. Additionally, a better understanding of the key features of forming carbon based hybrids (including by functionalizing the carbon surface) will the development of novel protocols that can generate ideas for more affordable and reliable approaches to the production of advanced hybrid materials.

## Author Contributions

EK, PK, and MS conceptualized the manuscript and completed the text. DA and AN drafted the manuscript.

## Conflict of Interest

The authors declare that the research was conducted in the absence of any commercial or financial relationships that could be construed as a potential conflict of interest.
